# nNOS splice variants differentially regulate myofilament function but are dispensable for intracellular calcium and force transients in cardiac papillary muscles

**DOI:** 10.1371/journal.pone.0200834

**Published:** 2018-07-20

**Authors:** W. Glenn L. Kerrick, Yuanyuan Xu, Justin M. Percival

**Affiliations:** 1 Department of Physiology and Biophysics, University of Miami Miller School of Medicine, Miami, Florida, United States of America; 2 Department of Molecular and Cellular Pharmacology, University of Miami Miller School of Medicine, Miami, Florida, United States of America; Cinvestav-IPN, MEXICO

## Abstract

Cardiac muscle expresses three neuronal nitric oxide synthase (nNOS) splice variants: nNOSα, nNOSμ and nNOSβ. The functions of these nNOS splice variants in cardiac muscle, particularly myofilament-associated nNOSβ are unclear. To decipher cardiac nNOS splice variant function we investigated myofilament function and intracellular calcium and force transients in demembranated and intact papillary muscles from two lines of nNOS knockout mice. The first line (KN1) lacks nNOSα and nNOSμ. The second line (KN2) lacks active nNOSα, nNOSμ and nNOSβ. Demembranated KN1 papillary muscles exhibited reduced myofilament ATPase activity (-35%) and specific force (-10%) relative to controls. Demembranated KN2 muscles exhibited a smaller decrease in myofilament ATPase activity (-21%), but a greater reduction in specific force (-26%) relative to controls. Myofilament calcium sensitivity in demembranated KN1 and KN2 papillary muscles was similar to controls. Thus, papillary muscle-expressed nNOS splice variants are necessary for control levels of myofilament ATPase activity and force generation, but dispensable for myofilament calcium sensitivity. The greater reduction in myofilament ATPase relative to specific force in KN1, but not KN2 muscle, reduced the energy cost of muscle contraction, suggesting that nNOSβ increased the energetic efficiency of contraction in the absence of nNOSμ and nNOSα. Analyses of intact KN1 and KN2 papillary muscles showed that both intracellular calcium transients and their evoked force transients were similar to controls at stimulation frequencies between 1 and 3 Hz. Therefore, nNOS was dispensable for baseline excitation-contraction coupling. In summary, these data suggest that nNOS splice variants differentially regulate myofilament function, but not baseline calcium handling in papillary muscles. More importantly, they suggest that nNOSβ is a novel modulator of myofilament function, and ultimately the energetic efficiency of cardiac papillary muscle contraction.

## Introduction

Neuronal nitric oxide synthase (nNOS) splice variants encoded by the *NOS1* gene are an important source of cardioprotective nitric oxide (NO) in the heart [1–4]. Cardiac muscle cells express three nNOS splice variants (nNOSα, nNOSμ and nNOSβ) at distinct subcellular locations. nNOSα and/or nNOSμ associate with the sarcoplasmic reticulum, while nNOSβ associates with myofilaments [5, 6]. These distinct localizations suggest isoform-specific functions for nNOS splice variants that remain to be deciphered. The functions of nNOSα and nNOSμ in cardiac muscle have received significant attention; however, the functions of nNOSβ are unknown.

**Fig 1:**
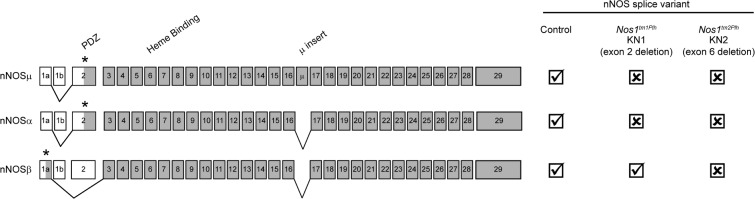
Exon structure of cardiac muscle expressed nNOS splice variants and their expression profile in the nNOS null mouse models used in this study. Coding exons of each splice variant are gray numbered boxes. Exon 2 encodes the PDZ (PSD95/Dlg1/ZO1) protein-protein interaction domain. Exon 6 encodes the heme binding domain essential for nitric oxide synthesis. Exon sequences that form unique 5’ untranslated sequences are white. Asterisks mark translation initiation sites. nNOS splice variant expression in control and murine KN1 and KN2 nNOS knockout models are shown on the right. A tick mark indicates expression. A cross mark indicates absence of expression and/or activity. This study employs two nNOS knockout models: first knockout of nNOS (KN1, exon 2 deletion) and second knockout of nNOS (KN2, exon 6 deletion). KN1 and KN2 mice have distinct isoform expression profiles. KN1 mice lack nNOSα and nNOSμ, but still express nNOSβ. KN2 mice do not express any active nNOS splice variants.

The functions of nNOSα and nNOSμ have been determined primarily from studies of “nNOS knockout” or *NOS1*^*-/-*^ mice that lack nNOSα and nNOSμ expression. *NOS1*^*-/-*^ mice are also called KN1 (knockout of nNOS 1) (Fig 1) [7–9]. In general, nNOSα and/or nNOSμ perform cardioprotective roles under conditions of stress [1–4]. However, there is no consensus on some aspects of nNOS function due to divergent findings by different studies. For example, one study reported that KN1 mice exhibited normal baseline ventricular function [10]. In contrast, a separate study of KN1 mice reported improved baseline ventricular function indicated by increased ejection fraction [11]. In agreement, cardiomyocyte-specific nNOSα overexpression or viral delivery of nNOSα to the KN1 myocardium impaired cardiac contractility [12, 13]. Another example of the lack of consensus comes from divergent findings regarding the role of nNOS in modulating myocardial contractile reserve. KN1 mice can exhibit impaired or enhanced β-adrenergic inotropic responses [10, 14, 15].

In vitro studies of nNOS function in cardiomyocytes isolated from KN1 mice have also yielded inconsistent results. Baseline Ca^2+^ transients, and sarcomere shortening can be unaffected, decreased or increased in cardiomyocytes [10, 11, 15, 16]. In addition, KN1 cardiomyocytes exhibit both increased and reduced Ca^2+^ leak from the sarcoplasmic reticulum [17, 18]. The reasons for some of these divergent results are beginning to emerge. For example, the regulation of Ca^2+^ leak by nNOS appears to be strongly temperature dependent so that temperature differences could account for differences in Ca^2+^ leak between studies [19]. However, uncertainty remains regarding many of the myocardial functions of nNOS splice variants.

In addition to temperature, variability in findings between studies of KN1 mice could also come from differences in mouse genetic background, and/or compensatory changes in nNOSβ activity. For example, KN1 mice in a mixed B6:129 background show reduced skeletal muscle fatigue resistance; however, KN1 mice on a congenic C57BL/6J background show normal fatigue resistance [9, 20, 21]. Background is also an important determinant of the cardiovascular phenotypes of mice lacking NO-sensitive soluble guanylyl cyclase, which is an important effector for nNOS. In addition, KN1 mice are not strictly nNOS knockouts because they still express catalytically active nNOSβ and catalytically inactive nNOSγ [7, 22]. Importantly, KN1 mice can express nNOSβ at increased levels presumably to compensate for the loss of nNOSα and nNOSμ [22]. Thus, increased nNOSβ activity could confound the interpretation of KN1 phenotypes.

To circumvent the confounding influence of nNOS splice variants in KN1 mice, we have used a second nNOS knockout called KN2 (knockout of nNOS 2) that lacks all nNOS splice variant activity (Fig 1) [9]. These mice do not express any active nNOS splice variants because KN2 mice lack exon 6 of the *NOS1* gene, which is essential for the synthesis of NO. Therefore, tissues from KN2 mice lack all nNOS (nNOSαnNOSβ nNOSμ) activity [8, 9]. Because nNOSβ knockout mice are currently not available, the functions of nNOSβ can be inferred indirectly by comparing the phenotypes of KN1 and KN2 mice that differ by nNOSβ activity [9].

Therefore, because the roles of nNOS in cardiac muscle remain unclear, or unknown in the case of nNOSβ, we investigated myofilament function and excitation-contraction coupling in intact and chemically demembranated ventricular papillary muscles isolated from adult KN1 and KN2 mice in congenic C57BL/6J backgrounds. Our data suggest that nNOS splice variants are necessary for normal myofilament function and differentially regulate myofilament ATPase activity and specific force (force output normalized to muscle area), but not myofilament Ca^2+^ sensitivity. We provide indirect evidence that myofilament-associated nNOSβ may regulate myofilament function in order to decrease the energy cost of muscle contraction in the absence of nNOSα and nNOSμ. Changes in myofilament function were not accompanied by changes in excitation-contraction coupling because nNOS inhibition had no impact on intracellular Ca^2+^ and force transients (force output normalized to muscle area) in KN1 and KN2 papillary muscles at different stimulation frequencies. Therefore, nNOS splice variants are dispensable for baseline excitation-contraction coupling in cardiac papillary muscle. Taken together, these findings suggest important roles for nNOS splice variants, particularly nNOSβ, in the regulation of the function of myofilaments that form part of the contractile machinery in cardiac muscle cells.

## Results

**Fig 2:**
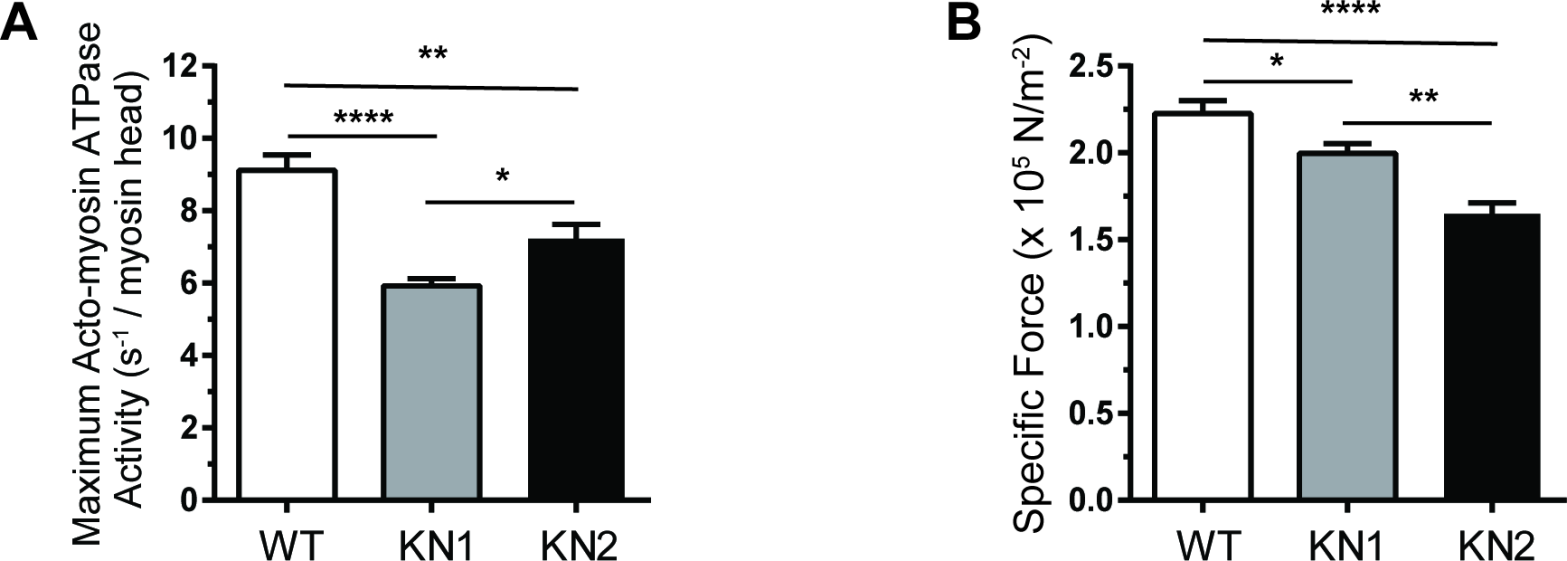
nNOS inhibition decreases maximum myofilament ATPase activity and specific force output in demembranated papillary muscle fibers. (A) Maximal myofilament acto-myosin ATPase activities in demembranated papillary muscles from WT, KN1 and KN2 mice. Maximum ATPase activities were significantly decreased in KN1 and KN2 papillary muscles relative to WT controls. ATPase activities in KN2 papillary muscles were intermediate between KN1 and wild type. (B) Demembranated papillary muscles from KN1 and KN2 showed a significant decrease in specific force output relative to WT controls. n = 10–12 mice per group (2–4 fibers per mouse). One factor ANOVA, * p < 0.05, ** p < 0.01, **** p < 0.001.

To determine if nNOS splice variants differentially regulate myofilament function in cardiac muscle cells, we first investigated maximal myofilament ATPase activity in chemically demembranated papillary muscles from KN1 and KN2 mice (Fig 2). Maximal myofilament ATPase activity decreased significantly by 35% in papillary muscles from KN1 mice compared to wild type controls (Fig 2A). Myofilament ATPase activity was reduced to a lesser degree (-21%) in KN2 mice, suggesting that nNOSβ may suppress maximal myofilament ATPase activity in muscles lacking nNOSα and nNOSμ. Reduced ATPase activity was accompanied by significant reductions in specific force (maximum force normalized to muscle area) in both KN1 and KN2 papillary muscles relative to controls (Fig 2B). Specific force was decreased to a greater degree in KN2 than KN1 papillary muscles compared to controls indicating a role for nNOSβ in modulating cardiac myofilament force output. Taken together, these data suggest that nNOSβ regulates myofilament function in cardiac muscle cells.

**Fig 3:**
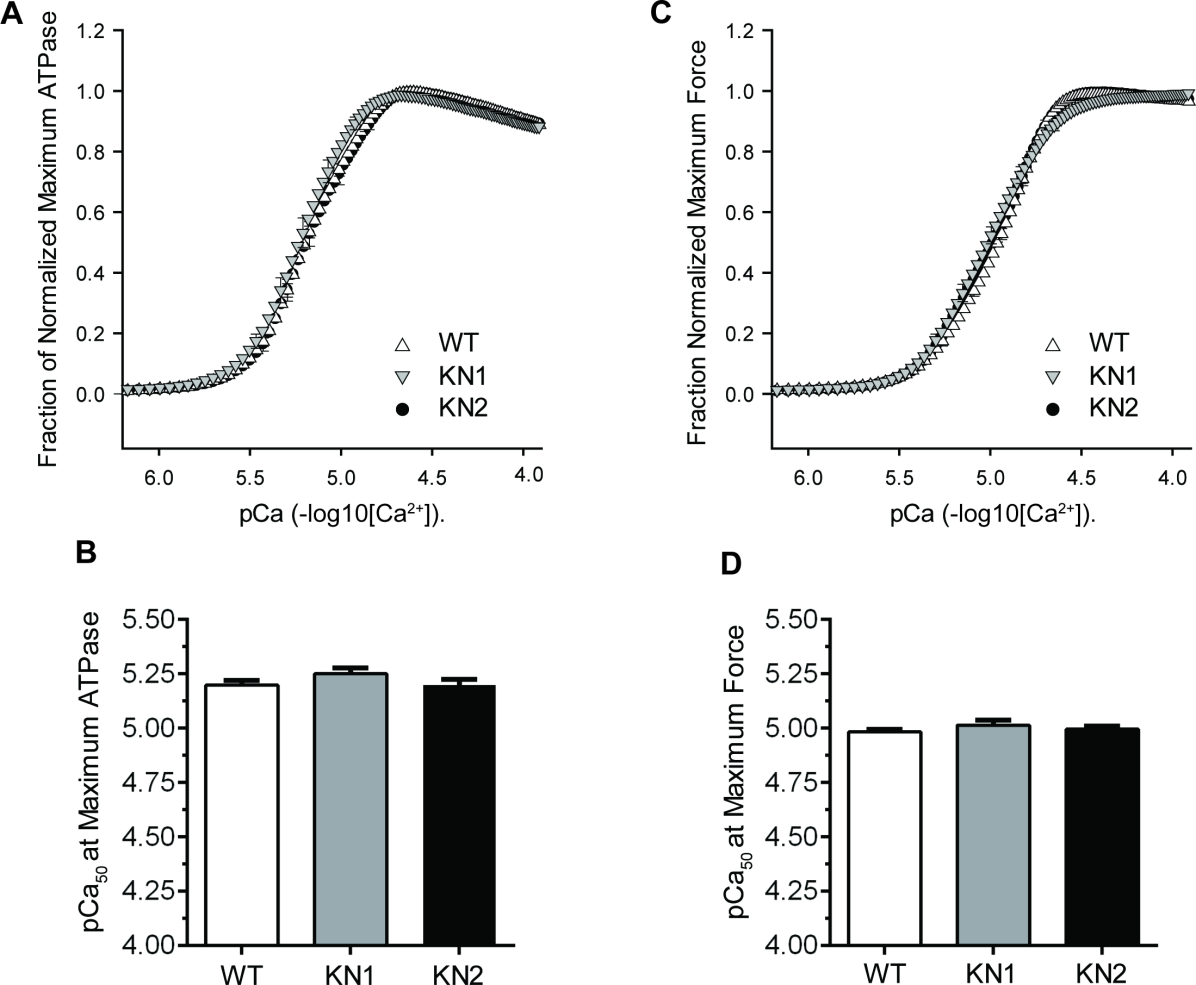
nNOS inhibition has no impact on the Ca^2+^ sensitivity of myofilament ATPase activity or force output in papillary muscle fibers. (A) Dependence of myofilament ATPase activity on Ca^2+^ concentrations in demembranated papillary muscles from WT, KN1 and KN2 mice. Normalized maximum ATPase activities were plotted against Ca^2+^ (pCa = -log_10_[Ca^2+^]). KN1, KN2 and WT control papillary muscles exhibited similar Ca^2+^ concentrations required for the activation of ATPase activity. (B) Papillary muscles from WT, KN1 and KN2 mice showed similar Ca^2+^ concentrations required for half maximal activation of ATPase activity (pCa_50_). (C) Dependence of demembranated papillary muscle force output on Ca^2+^ in WT, KN1 and KN2 mice. nNOS inhibition had no impact on the force-Ca^2+^ relationship which was similar between KN1, KN2 and wild type controls. (D) Papillary muscles from WT, KN1 and KN2 mice showed similar Ca^2+^ concentrations required for half maximal force output (pCa_50_). n = 10–12 mice per group (2–4 fibers per mouse).

To investigate how nNOS inhibition impairs myofilament function in demembranated KN1 and KN2 papillary muscles, we tested if reduced myofilament Ca^2+^ sensitivity contributed to the deficits in myofilament ATPase activity and specific force (Fig 3). Myofilament ATPase activity was similar at all Ca^2+^ concentrations (pCa^2+^) between wild type, KN1 and KN2 papillary muscle fibers (Fig 3A). Accordingly, the pCa^2+^ concentration required for half-maximal myofilament ATPase (pCa^2+^_50_) was similar between wild type, KN1 and KN2 demembranated papillary muscles (Fig 3B). Therefore, these data indicate that nNOS does not regulate myofilament ATPase activity through the modulation of myofilament sensitivity to Ca^2+^.

We next investigated if the reductions in specific force in demembranated KN1 and KN2 papillary muscles were due to impaired myofilament sensitivity to Ca^2+^ by determining the force-pCa^2+^ relationship (Fig 3C). The force-pCa^2+^ relationships in demembranated papillary muscles from wild type, KN1 and KN2 mice were unaffected because normalized force output at all Ca^2+^ concentrations was similar between wild type, KN1 and KN2 muscles (Fig 3C). Accordingly, the pCa^2+^_50_ for activation of normalized maximal force were similar between WT, KN2 and KN1 papillary muscles. Therefore, these data suggest that cardiac muscle nNOS modulates specific force output without influencing myofilament Ca^2+^ sensitivity, and that nNOS is dispensable for normal baseline myofilament sensitivity to Ca^2+^.

**Fig 4:**
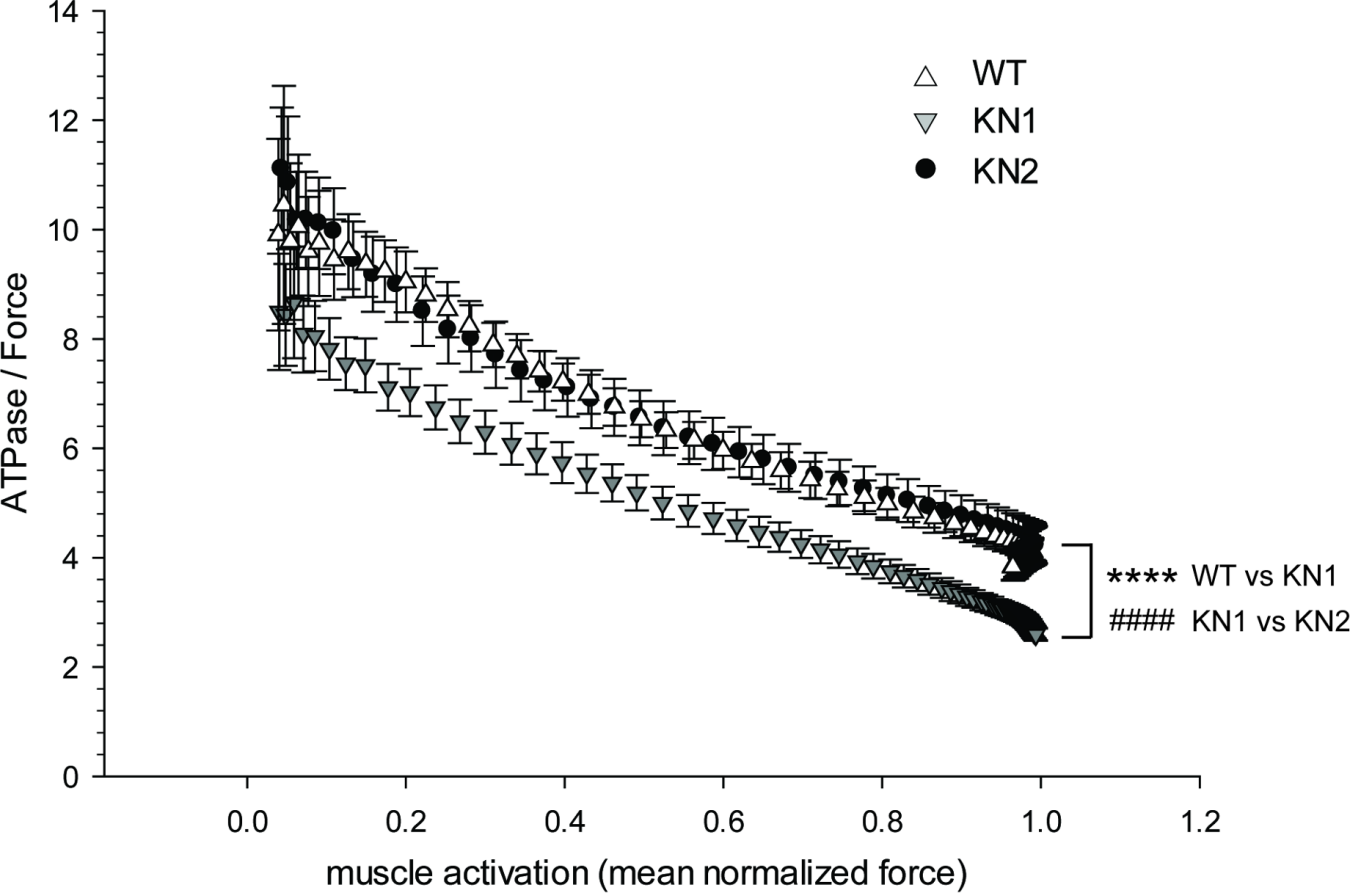
KN1 mice exhibit a reduced energy cost of muscle contraction. The energetic cost (maximal ATPase activity divided by force output) of muscle contraction was calculated at different muscle fractional activation states. Muscle activation is normalized muscle force output over the range of Ca^2+^ concentrations in Fig 3. Demembranated papillary muscles from KN1 mice exhibited a lower energy cost of contraction than wild type controls. KN2 papillary muscles exhibited a similar energy cost of contraction to wild type controls. n = 10–12 mice (2–4 fibers per mouse). Two factor ANOVA, ***** p < 0.001 WT versus KN1, #### p < 0.001 KN1 versus KN2.

The distinct reductions in myofilament ATPase activity and specific force in KN1 and KN2 muscles could differentially affect the energetic cost of cardiac muscle contraction. To test this possibility we calculated the energetic cost (maximal ATPase activity divided by maximal force output) of muscle contraction at different fractional levels of Ca^2+-^dependent muscle activation in demembranated KN1 and KN2 papillary muscles (Fig 4). The energy cost of cardiac muscle contraction was significantly lower in KN1 papillary muscles at most muscle activation states compared with wild type controls (Fig 4). Note that in low muscle activation states, the variability in the ATPase/force ratio is predictably high due to poor signal to noise ratios inherent to the approach. In contrast, the energetic cost of muscle contraction in KN2 papillary muscles was similar to wild type control muscles (Fig 4). These data suggested that the decreased energetic cost of Ca^2+^ activated muscle contraction in KN1 papillary muscles resulted from a possible increase in nNOSβ activity, and not from the loss of nNOSα and nNOSμ. Taken together, these studies provide evidence suggesting that nNOSβ regulates the energetic efficiency of cardiac muscle contraction through the modulation of myofilament function.

**Fig 5:**
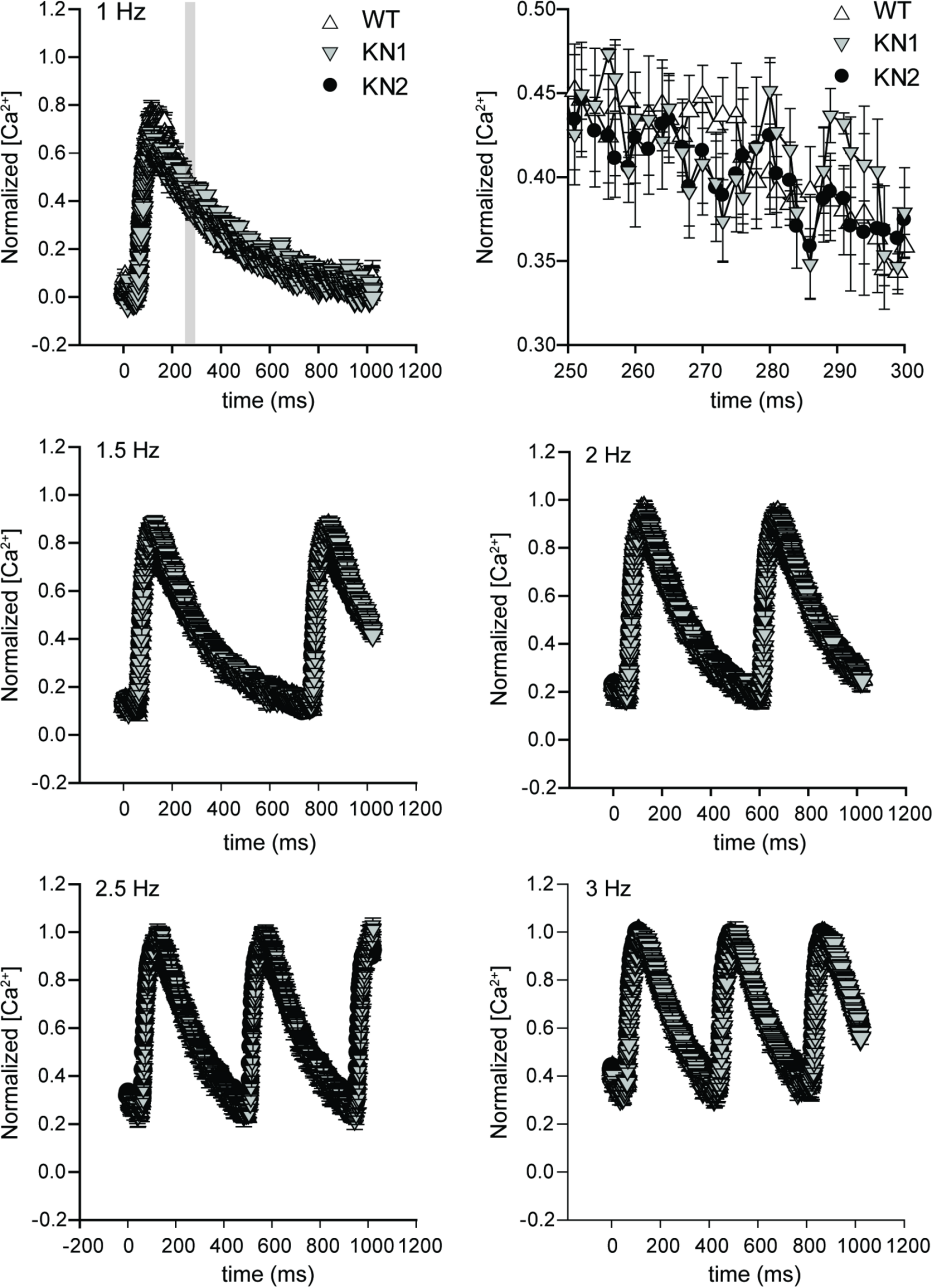
nNOS is dispensable for intracellular Ca^2+^ transients in intact cardiac papillary muscles. The impact of nNOS splice variant deficiency on Ca^2+^ transients was determined in intact papillary muscles loaded with Fura-2 Ca^2+^ indicator paced at different stimulation frequencies. Simultaneous measurements of intracellular Ca^2+^ (here) and force (Fig 6) were recorded when force reached a steady state following changes in stimulus frequencies. For each stimulation frequency, intracellular Ca^2+^ transients were averaged and plotted as a function of time. Ca^2+^ transients in intact papillary muscles from KN1 and KN2 mice were indistinguishable from wild type controls, and from each other, at all stimulation frequencies. The top right panel shows a high magnification image of intracellular Ca^2+^ concentrations between 250–300 ms (gray box in top left panel-1 Hz stimulation) in intact papillary muscles from wild type, KN1 and KN2 mice to highlight extensive data overlap. n = 4–6 mice per group.

**Fig 6:**
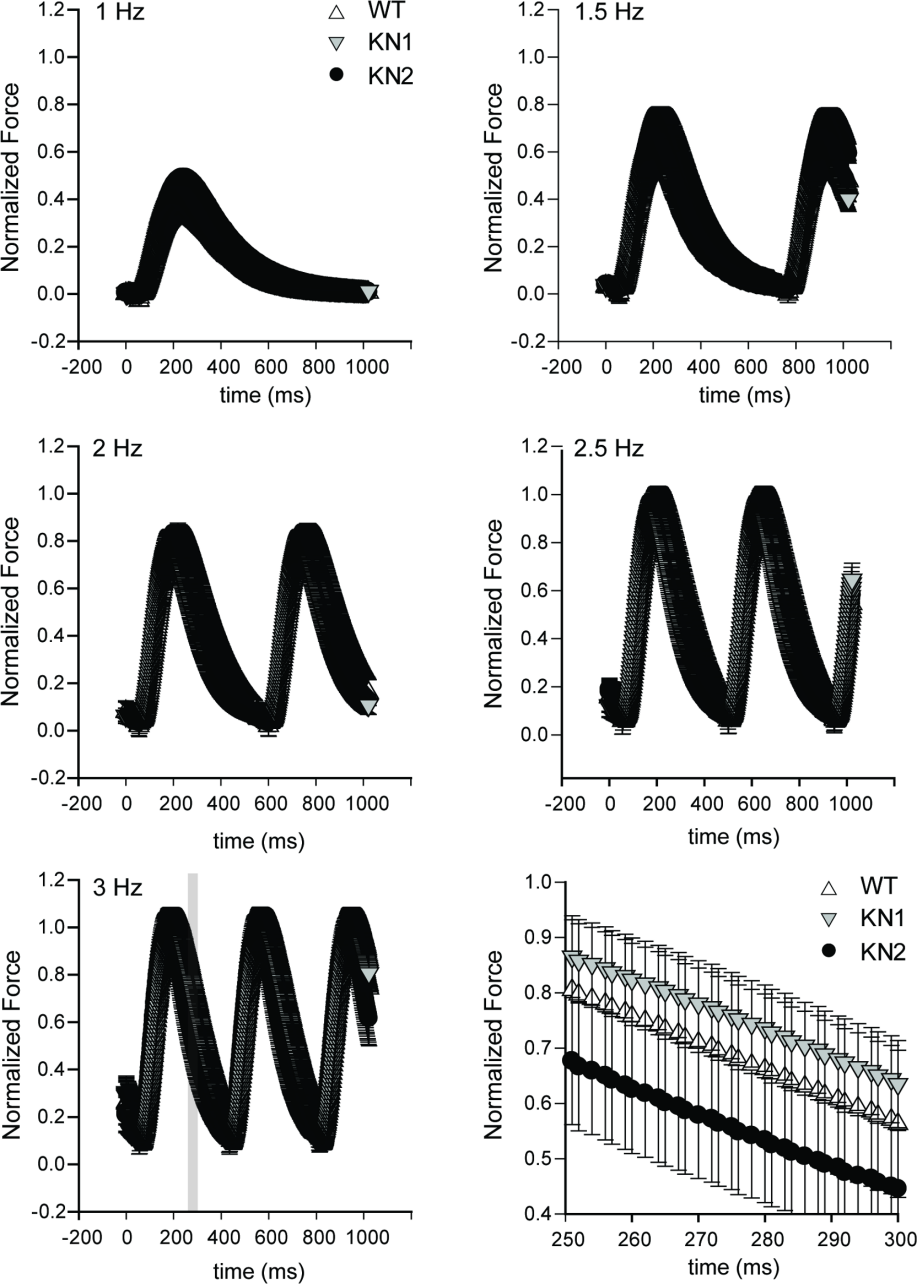
nNOS inhibition has no impact on force responses to Ca^2+^ transients in intact papillary muscles. The impact of nNOS splice variant deficiency on the normalized force (fraction of maximum) response to Ca^2+^ transients (shown in Fig 5) was determined in intact papillary muscles at different stimulation frequencies. Normalized force output from intact papillary muscles from KN1 and KN2 mice was indistinguishable from wild type controls, and from each other, at stimulation frequencies between 1 and 3 Hz. The bottom right panel shows a high magnification image of normalized force output between 250–300 ms (gray box in bottom left panel-3 Hz stimulation) in intact papillary muscles from wild type, KN1 and KN2 mice to highlight extensive data overlap. n = 4–6 mice per group.

We next investigated whether nNOS splice variant inhibition affected Ca^2+^ handling and contractility in intact (non-demembranated) KN1 and KN2 papillary muscles by simultaneously measuring intracellular Ca^2+^ transients (which reflect sarcoplasmic reticulum Ca^2+^ release and uptake) and their evoked force responses. Intact KN1 and KN2 papillary muscles were loaded with Fura-2 Ca^2+^ indicator and Ca^2+^ and force transients were simultaneously measured at different stimulation frequencies (1–3 Hz) using the Guth muscle research system [23, 24]. Normalized intracellular Ca^2+^ transients in intact papillary muscles from wild type controls, KN1 and KN2 mice were indistinguishable from one another at stimulation frequencies between 1 and 3 Hz (Fig 5). Similarly, the contractile force responses to the Ca^2+^ transients in intact KN1 and KN2 papillary muscle fibers did not differ from each other or from wild type controls at stimulation frequencies between 1 and 3 Hz (Fig 6). Therefore, these data demonstrate that the force-frequency and Ca^2+^-frequency relationships are preserved in intact KN1 and KN2 papillary muscles, and provide compelling evidence that nNOS splice variants do not play an important role in baseline excitation-contraction coupling in intact ventricular papillary muscles.

## Discussion

The primary finding of this study is that nNOS splice variants differentially regulated myofilament function in ventricular papillary muscles. All three nNOS splice variants were necessary for control levels of myofilament ATPase activity and specific force output. However, KN2 muscles exhibited a greater reduction in specific force than KN1 muscles suggesting that nNOSβ regulates myofilament function to promote force generation. Interestingly, our data suggested that nNOSβ also contributed to the deficits in ATPase activity in KN1 muscles and was responsible for the improved energy cost of Ca^2+^ activated muscle contraction in KN1 muscles. These findings suggest that nNOSβ contributes to the cardiac phenotype of KN1 mice. nNOSβ was previously reported to be associated with myofilaments in cardiac muscle tissue [6]. Our data suggest that this association may enable nNOSβ to regulate myofilament function, and ultimately the energetic efficiency of cardiac muscle contraction.

nNOS inhibition did not impact all aspects of myofilament function because myofilaments Ca^2+^ sensitivity was unaffected in both KN1 and KN2 papillary muscles. Therefore our data suggest that low levels of NO synthesized by endogenous nNOS splice variants may not impact baseline myofilament Ca^2+^ sensitivity. This finding contrasts with a report that addition of exogenous S-nitrosothiols to demembranated papillary muscles impaired myofilament Ca^2+^ sensitivity [25]. These findings are not mutually exclusive. It is possible that under some conditions, such as pathophysiological states characterized by the loss of the tight spatio-temporal control of NO synthesis and levels, that the production of high concentrations of NO at the wrong time or place could reduce myofilament sensitivity to Ca^2+^.

How might nNOS inhibition impair myofilament ATPase activity and specific force? Reduced myofilament Ca^2+^ sensitivity is an improbable cause because it was unaffected in KN1 and KN2 papillary muscles. In addition, preservation of normal myofilament Ca^2+^ sensitivity argues against functional differences in Ca^2+^ regulatory proteins (such as troponin and/or myosin) in KN1 and KN2 papillary muscles. Reductions in myofilament ATPase can result from a decrease in the number of myosin cross-bridges between thin actin and thick myosin filaments, a decrease in the cross-bridge formation rate, both of which could result from reduced myosin protein levels. Therefore, the impaired myofilament ATPase activity in KN1 and KN2 papillary muscles could result from reductions in myosin cross bridge number, myosin cross bridge formation and/or myosin expression. Reductions in myosin cross-bridge number, and/or decreases in force per cross-bridge could also explain specific force deficits in KN1 and KN2 papillary muscles.

More importantly, the differences in the degree of impairment of myofilament ATPase activity and specific force between KN1 and KN2 papillary muscles manifested in a significant difference in the energy cost of muscle contraction. KN1 papillary muscles showed a significantly lower energy cost of contraction across a wide range of Ca^2+^-dependent muscle activation states. In contrast, the energy cost of contraction in KN2 papillary muscles was the same as wild type controls suggesting that nNOSβ was driving the improved energetic efficiency of contraction in KN1 muscles. The energy cost of contraction is described by the following equation: energy cost = ATPase/Specific Force = g_app_ (L/(F_avg_L_1/2_)) where g_app_ is rate of myosin cross-bridge dissociation, L is muscle fiber length which is constant under these experimental conditions, F_avg_ is the average force per cross-bridge, and L_1/2_ is half of the sarcomere length [26, 27]. The continuous decrease in energy cost with increasing muscle activation is explained by nearest neighbor cycling myosin cross-bridge effects on myosin ATPase where greater numbers of nearest neighbor cross-bridges attachments progressively decrease myosin ATPase activity [28]. For KN1 papillary muscles, the maximum myofilament ATPase activity decreases more than the Ca^2+^-activated specific force. This is not the case for KN2 muscles where myofilament ATPase and specific force decrease proportionally resulting in no overall change relative to controls. Therefore, the decrease in energy cost in KN1 papillary muscles is due to either a decrease in g_app_, and/or an increase in F_avg_, both which are determined by the myosin. Therefore, in KN1 papillary muscles, nNOSβ may modulate myofilament function by decreasing myosin ATPase activity and/or increasing force produced per myosin cross bridge attachment; thereby increasing the energetic inefficiency of contraction at the same time.

These findings suggest nNOS splice variants differentially regulate myofilament function, and ultimately the energy cost of contraction, by controlling myosin ATPase activity and the number of myosin heads interacting with actin filaments to form cross-bridges. Previous studies support myosin as an important target for NO in cardiac muscle cells. α-myosin heavy chain and myosin essential light chain can be S-nitrosated on cysteines at multiple sites by exogenous nitrosothiols [25]. Remarkably, S-nitrosation can convert myosin from a high speed-low force motor to a low speed-high force motor [29]. Also as mentioned earlier, application of exogenous nitrosothiols can decrease myofilament ATPase activity in papillary muscles [25]. Collectively, these findings support myosin as a key target of NO, particularly NO synthesized by endogenous nNOS splice variants, in cardiac muscle cells.

However, the changes in myofilament function in demembranated KN1 and KN2 papillary muscles were insufficient to affect the time course and shape of Ca^2+^ and force transients in intact KN1 or KN2 papillary muscles. Indeed, our data suggest that nNOS is dispensable for normal Ca^2+^ handling and contraction in cardiac papillary muscles at baseline. Previous studies of cardiomyocytes from KN1 mice have yielded divergent results regarding the roles of nNOSμ and/or nNOSα in contraction and Ca^2+^ handling. Previous studies showed that baseline shortening and Ca^2+^ transient amplitudes were normal in cardiomyocytes isolated from KN1 mice on a C57Bl6/J background [10, 30]. In contrast, cardiomyocytes isolated from KN1 mice on a mixed background exhibited increased basal shortening and Ca^2+^ transient amplitude [11, 14]. Our studies of papillary muscles from KN1 mice (congenic C57BL/6 background) do not support a role for nNOSμ and/or nNOSα in baseline Ca^2+^ handling and contraction. They suggest that intracellular Ca^2+^ transient behavior (the timed rise and fall in intracellular Ca^2+^ due to SR release and reuptake, respectively), and contractile force responses to increased Ca^2+^ transients are normal in intact KN1 papillary muscles regardless of stimulation frequency. Our data agree with the findings in KN1 cardiomyocytes of Barouch *et al*. and Khan *et al*. that nNOSα and/or nNOSμ are dispensable for normal baseline Ca^2+^ handling and contractility [10, 30].

It is important to note three salient technical factors that are important when comparing our study to previous reports. First, we performed our experiments in intact papillary muscle cells and not isolated cardiomyocytes. Second, we performed our studies at temperatures less than 30°C where nNOS-regulated Ca^2+^ leak from the sarcoplasmic reticulum is not a major consideration [19]. Third, genetic inhibition of nNOS could affect muscle cell excitation-contraction at stimulation frequencies greater than the 3 Hz used here as reported previously [30]. We cannot test this possibility because the stimulation of papillary muscles at frequencies greater than 3 Hz results in non-uniform steady state twitches. This is a technical limitation of our approach. It is also important to note that our data do not preclude the possibility that nNOS splice variants expressed in other cell types may regulate muscle cell contractility. For example, nNOS expressed in neurons of the autonomic and sympathetic nervous systems may play a role in regulating myocardial contractility under physiological or pathophysiological conditions. Nonetheless, an increasing amount of evidence supports the contention that nNOSμ and/or nNOSα are dispensable for baseline cardiac muscle excitation-contraction in mice on a congenic C57BL/6 background.

As mentioned earlier, differences in temperature and/or in mouse genetic background could contribute to differences between studies of nNOS function in cardiac muscle. In addition, disparities between studies could also come from differences in compensatory changes in nNOS splice variant activity in KN1 mice. KN1 mice in a mixed background express normal or higher levels of fully catalytically active nNOSβ protein, particularly in neurons, presumably to compensate for the loss of nNOSα and nNOSμ [22]. It is not clear if nNOSβ upregulation also occurs in congenic C57BL/6J mice. In addition, it is important to note that this study did not demonstrate a cause and effect relationship between changes in myofilament function and nNOSβ expression and activity. Nonetheless, our data suggests that nNOSβ modulates cardiac myofilament function in KN1 papillary muscles lacking nNOSα and nNOSμ. Specifically, nNOSβ contributed to the deficits in myofilament ATPase activity and improved energy cost of contraction in KN1 papillary muscles. These findings highlight an important caveat when interpreting KN1 cardiac phenotypes, which in some circumstances could result from the loss of nNOSμ and/or nNOSα function and/or changes in nNOSβ activity.

## Conclusions

In conclusion, our study provides compelling evidence that nNOS splice variants differentially regulate myofilament function in cardiac muscle cells. This evidence is consistent with the association of nNOSβ with myofilaments and with reports that NO exerts direct regulatory effects on myofilament proteins such as myosin. Our data also bolster the argument that nNOS isoforms do not play an important role in baseline Ca^2+^ handling and contraction in cardiac muscle cells from C57BL/6J mice. Importantly, we provide new clues about the functions of nNOSβ in cardiac muscle by providing evidence that nNOSβ is a novel regulator of myofilament function, specifically ATPase activity and specific force output that ultimately reduce the energy cost of cardiac muscle contraction. These findings significantly expand current understanding of nNOS splice variant function in the heart.

## Materials and methods

### Mouse models

Two *NOS1* gene-targeted mouse lines were used to study nNOS function. The first was the nNOS knockout 1 (KN1 or *NOS1*^-/-^) line generated by targeted deletion of exon 2 [7, 9]. The second mouse line was the nNOS knockout 2 (KN2) generated by targeted deletion of exon 6, which inhibits the activity of all nNOS splice variants [8, 9]. Both KN1 and KN2 mouse lines are on congenic C57BL/6J backgrounds. Equal numbers of two-month-old male and female KN1 and KN2 mice and wild type littermate controls were analyzed. The Institutional Animal Care and Use Committee of the University of Miami approved all experimental procedures performed on mice.

### Intact papillary muscle fiber studies

Papillary muscles were rapidly dissected from the right ventricle into oxygenated Krebs-Henseleit solution and mounted in the Guth Muscle Research System (Scientific Instruments GmbH, Heidelberg, Germany) [23, 24]. Papillary fibers were loaded with 5 μM of Ca^2+^ indicator Fura-2 acetoxymethyl (AM) ester for 1 hour in an oxygenated Krebs-Henseleit solution containing 0.5% Cremophor detergent. After loading, the muscle was rinsed, then continuously perfused with Krebs-Henseleit solution. All experiments were performed between 21 and 24 ^o^C. Muscles were paced at different stimulation frequencies by tweezers attached to the ends of the papillary muscles. Once the preparation was mounted in the Guth System, muscles were adjusted to optimum length (Lo) where maximum twitch force was obtained. Ca^2+^ transients were measured using the ratio of Fura-2 AM 340/380 fluorescence emssions. Background fluorescence from 340 and 380 nm excitation of the unloaded preparation was subtracted from 340 and 380 nm emission signals from the Fura-2 AM loaded preparation. Fura-2 AM 340/380 fluorescence ratios were used to calculate relative changes in the intracellular [Ca^2+^] transients as described previously [31]. Changes in force and fluorescence were simultaneously recorded with Guth Muscle Research System software.

### Demembranated papillary muscle fiber studies

60–70 μm diameter papillary muscle fiber strips were dissected from excised hearts into 4 ^o^C relaxing solution (85 mM K^+^, 2 mM MgATP, 1 mM Mg^2+^, 7 mM EGTA (pH 7.0), with propionate as the major anion). Papillary fiber strips were then chemically demembranated with 1% Triton X-100 for 30 min and processed immediately without glycerinating [31]. Chemically demembranation or skinning eliminates the effects of membrane systems including the sarcolemma, T-tubules, sarcoplasmic reticulum, and mitochondrial on force development and ATPase activity and enables precise control of Ca^2+^ concentrations. Demembranated fibers were mounted in a Guth muscle research system and muscle length stretched to 10% of slack length. Fiber cross-sectional area was calculated from fiber in diameter determined microscopically with the assumption that fiber geometry is circular. Myofilament ATPase rate was measured using the NADH fluorescence method [23] as described previously [23, 31, 32]. Papillary fibers were subjected to an increasing Ca^2+^ gradient produced by mixing the relaxing (pCa 9) and contracting (pCa 3.4) solutions as previously described [33]. Both solutions contained 85 mM K+, 2 mM MgATP^2−^, 1 mM Mg^2+^, 7 mM EGTA, 5 mM phosphoenolpyruvate, 100 U/ml pyruvate kinase (PK), 0.4 mM NADH, 140 U/mL lactate dehydrogenase, ionic strength 150 mM (pH 7.0), and propionate. Fresh NADH solution was added to the reaction cuvette every 20 seconds. The decrease in NADH concentration was determined by decreased fluorescence emission at 450 nm. The slope of the linear decrease in NADH concentration was used to calculate the ATPase rate. The Ca^2+^ concentration gradient was calibrated by use of the fluorescent Ca^2+^ indicator calcium green-2 (Invitrogen) [33]. Force development was measured using the force transducer of the Guth system and was determined simultaneously with ATPase measurements.

### Statistical analyses

Mean and standard error of the mean are reported in all figures. One factor ANOVA with Tukey’s multiple comparison tests was used to determine statistically significant differences in means between one variable in more than two groups. Two factor ANOVA with Tukey’s multiple comparison tests was used to determine statistically significant differences between means in studies with two variables. Statistical analyses were performed using Prism v 6.07 (Graphpad Software, Inc.). *p* values less than 0.05 were considered significant.
